# Formate-induced CO tolerance and methanogenesis inhibition in fermentation of syngas and plant biomass for carboxylate production

**DOI:** 10.1186/s13068-023-02271-w

**Published:** 2023-02-17

**Authors:** Flávio C. F. Baleeiro, Lukas Varchmin, Sabine Kleinsteuber, Heike Sträuber, Anke Neumann

**Affiliations:** 1 Department of Environmental Microbiology, Helmholtz Centre for Environmental Research – UFZ, Leipzig, Germany; 2 Technical Biology, Institute of Process Engineering in Life Science, Karlsruhe Institute of Technology – KIT, Karlsruhe, Germany

**Keywords:** Mixotrophy, Volatile fatty acids, Medium-chain carboxylic acids, Formic acid, Chain elongation, Microbiome, Carbon monoxide, Ethene, Hexanoic acid, Lactic acid bacteria

## Abstract

**Background:**

Production of monocarboxylates using microbial communities is highly dependent on local and degradable biomass feedstocks. Syngas or different mixtures of H_2_, CO, and CO_2_ can be sourced from biomass gasification, excess renewable electricity, industrial off-gases, and carbon capture plants and co-fed to a fermenter to alleviate dependence on local biomass. To understand the effects of adding these gases during anaerobic fermentation of plant biomass, a series of batch experiments was carried out with different syngas compositions and corn silage (pH 6.0, 32 °C).

**Results:**

Co-fermentation of syngas with corn silage increased the overall carboxylate yield per gram of volatile solids (VS) by up to 29% (0.47 ± 0.07 g g_VS_^−1^; in comparison to 0.37 ± 0.02 g g_VS_^−1^ with a N_2_/CO_2_ headspace), despite slowing down biomass degradation. Ethylene and CO exerted a synergistic effect in preventing methanogenesis, leading to net carbon fixation. Less than 12% of the electrons were misrouted to CH_4_ when either 15 kPa CO or 5 kPa CO + 1.5 kPa ethylene was used. CO increased the selectivity to acetate and propionate, which accounted for 85% (electron equivalents) of all products at 49 kPa CO, by favoring lactic acid bacteria and actinobacteria over *n*-butyrate and *n*-caproate producers. Inhibition of *n*-butyrate and *n*-caproate production by CO happened even when an inoculum preacclimatized to syngas and lactate was used. Intriguingly, the effect of CO on *n*-butyrate and *n*-caproate production was reversed when formate was present in the broth.

**Conclusions:**

The concept of co-fermenting syngas and plant biomass shows promise in three aspects: by making anaerobic fermentation a carbon-fixing process, by increasing the yields of short-chain carboxylates (propionate and acetate), and by minimizing electron losses to CH_4_. Moreover, a model was proposed for how formate can alleviate CO inhibition in certain acidogenic bacteria. Testing the fermentation of syngas and plant biomass in a continuous process could potentially improve selectivity to *n*-butyrate and *n*-caproate by enriching chain-elongating bacteria adapted to CO and complex biomass.

**Supplementary Information:**

The online version contains supplementary material available at 10.1186/s13068-023-02271-w.

## Background

Anaerobic mixed culture fermentation (anaerobic fermentation for short) enables the production of saturated monocarboxylates from cheap organic matter. The technology uses microbial communities as robust and self-regenerating biocatalysts to produce a spectrum of carboxylates with one to eight carbon atoms in their chain [1, 2]. Many technological aspects behind the production of these carboxylates are similar to those behind biogas production from anaerobic digestion [1]. Hence, anaerobic fermentation can take profit from part of the existing infrastructure and knowledge about anaerobic digestion while producing chemicals that are more valuable and energy-dense than biogas [3]. Carboxylates; in particular, those with longer carbon chains, can be extracted from fermentation broths with relative ease [4] and hold promise as a bio-based platform for the chemical and fuel industry [5].

Ultimately, the impact that anaerobic fermentation can have as a sustainable technology for transforming the economy is limited by the availability of organic feedstocks that are local, cheap, and biodegradable [6, 7]. Regardless if supplied by industrial waste streams, municipal organic waste, or plant biomass, the feasibility of the anaerobic fermentation plant is constrained by biomass cost, supply, and quality.

One way to make anaerobic fermentation more independent of organic feedstocks is by exploiting autotrophic and mixotrophic microorganisms that are highly efficient in consuming inorganic or both organic and inorganic substrates [8]. Due to the reductive acetyl-CoA pathway present in acetogenic bacteria, mixtures of H_2_, CO_2_, and CO (often called syngas) are one of the most promising inorganic co-substrates for anaerobic fermentation [9, 10]. In this pathway, CO_2_ is used as a terminal electron acceptor whereas H_2_ and CO are used to reduce electron carriers, such as NAD^+^ and Fd_ox_, acting as electron donors in the formation of acetyl-CoA.

Syngas can be produced directly via dry biomass gasification or mixtures of it can be blended by merging green technologies such as power-to-gas (supplying H_2_), carbon capture (supplying CO_2_), and valorization of industrial off-gases (supplying CO) [11]. Syngas fermentation opens many possibilities to combine such technologies for the production of value-added chemicals [12]. Among such possibilities, fermenting syngas and complex biomass in a one-pot process with mixed cultures is one of the most promising [5].

Recently, several studies have explored the use of microbial communities to produce carboxylates from syngas components in a one-pot process. They approached the topic by (1) innovative ways of supplying H_2_ [13, 14], (2) enrichment of microbial communities in mineral media with syngas [15–17], (3) co-fermentation of H_2_/CO_2_ (or H_2_/CO_2_/CO) with synthetic organic substrates [18–20], and (4) co-fermentation of H_2_/CO_2_ with organic waste streams [21]. In general, the presence of H_2_ and CO helped increase carboxylate yields and selectivity to medium-chain carboxylates. Yet, we found no studies with complete syngas mixtures (H_2_/CO_2_/CO) and plant biomass.

CO is expected to have a major impact on the plant biomass fermentation. Despite being a key substrate for acetogenic bacteria, carbon monoxide is a strong inhibitor of hydrogenases [22] and hence disrupts the electron transport chains of bacteria that rely on these enzymes. This is aggravated in fermentative bacteria relying on [Fe–Fe] hydrogenases [23], which are particularly sensitive to CO [24]. Yet, there are ways to overcome CO inhibition of fermentative bacteria. For instance, *Clostridium kluyveri* was less prone to CO inhibition when grown in co-culture with acetogens (e.g., *Clostridium autoethanogenum*) in bottles without shaking [25] or by inoculating it to a chemostat with an active carboxydotroph, able to maintain the CO concentrations in the liquid phase low [26, 27]. Besides, the presence of formate, a common extracellular metabolite in anaerobic microbial communities, allowed *Acetobacterium woodii* to tolerate up to 25 kPa CO [28].

In this study, we aimed to understand the main effects of syngas on the carboxylate production during the anaerobic fermentation of plant biomass using corn silage as a model feedstock. We focused on the syngas composition taking in consideration the double-edged role of CO in carboxylate production.

## Material and methods

### Batch cultivation

In an anaerobic chamber, 50 mL of anoxic mineral medium (preparation and composition are described in Additional file 1: Table S1, respectively) were added to 250 mL-serum bottles containing 5.5 g of fresh matter of corn silage each, resulting in a volatile solids (VS) concentration of 30 gVS L^−1^. All bottles contained at least the autochthonous microbial community of the corn silage as inoculum. Bottles additionally inoculated with a community adapted to syngas received cells from an enrichment reactor (see “Source of the adapted community”). To harvest the cells from the enrichment reactor, the fermentation broth was centrifuged at 4,816 × g and 4 °C for 10 min. The supernatant was discarded and the cells were washed and resuspended in the equal volume of anoxic mineral medium. Alternatively, the untreated broth from the enrichment reactor, which was rich in carboxylates, was either used instead of mineral medium or 10% of the medium was substituted with it (inoculation with 100% or 10% reactor broth). The serum bottles were closed with butyl rubber stoppers and sealed with aluminum crimps. All batch cultures were prepared in duplicates.

**Fig. 1:**
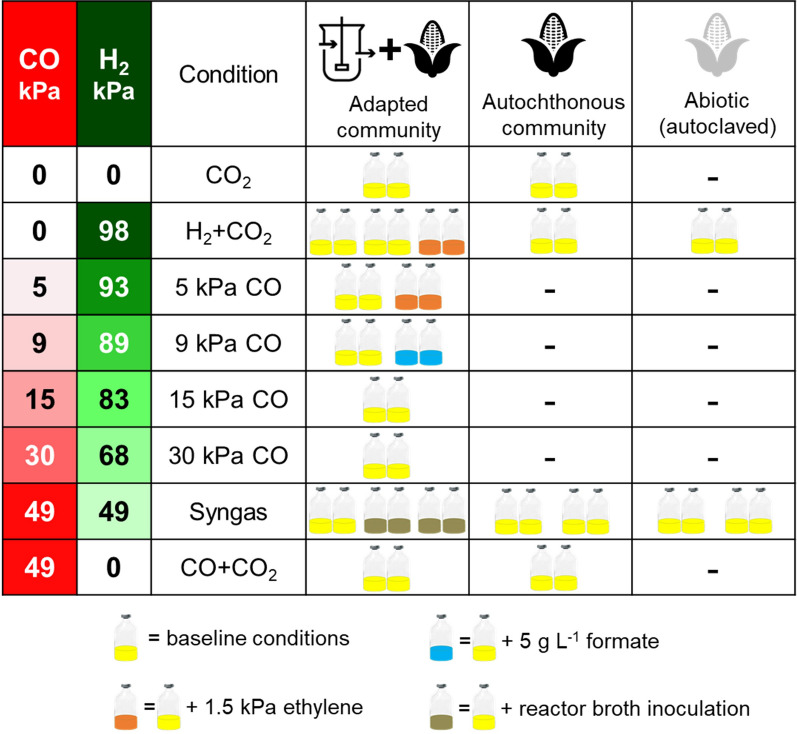
Summary of all conditions tested in batch cultures. Yellow bottles contained at least corn silage, mineral medium, and CO_2_. Additionally, CO and H_2_ were added as indicated. As special conditions, formate or ethylene was additionally added, or untreated reactor broth was used as source of the adapted community instead of washed cells

Different partial pressures of H_2_ and CO were applied (Fig. 1), resulting in a combined partial pressure of 98 kPa. This ensured a fixed amount of electron donors in the headspace. The only exceptions were the condition “CO_2_”, which contained neither CO nor H_2_, and the condition “CO + CO_2_^”^, which contained 49 kPa CO but no H_2_. All cultivation bottles initially contained 24 kPa CO_2_. N_2_ was used as a filling gas to reach the final pressure of 150 kPa (1.5 bar_a_). In two pairs of bottles, 3 mL ethylene was additionally added to the pressurized bottles to achieve 1.5 kPa ethylene at 0 kPa CO (98 kPa H_2_) and 5 kPa CO (93 kPa H_2_) (Fig. 1). In one pair of bottles, 0.2 mL of formic acid was added at 9 kPa CO (89 kPa H_2_) to reach a concentration of 5 g L^−1^ formate (Fig. 1). The pH was corrected with 4 M KOH immediately after formic acid addition. For abiotic controls, the sealed and pressurized bottles with corn silage and mineral medium were autoclaved for 20 min at 121 °C.

The batch cultures were incubated on a rotary shaker at 32 °C and 200 rpm. Fermentations were carried out at a pH value of 5.9 ± 0.5. Sampling of the liquid phase and pH correction with 4 M NaOH were done weekly. The pressure was monitored twice per week. When a pressure lower than 100 kPa was detected, the bottle was re-pressurized to 150 kPa with the applicable gas mixture. Before every pressurization, the headspace of the bottle was purged with 2.5 L of the corresponding gas mixture. By monitoring the chemicals in the gas and aqueous phase, little to no activity was detected in the abiotic controls (Additional file 1: Figure S1), and the amount of DNA extracted from cell pellets was too low for microbial community analysis. For practical reasons, the experiments were divided in three different batches lasting from 31 to 38 d. For information on how the experiments were divided in different batches and the exact duration of each batch see “Further details on the experimental setup” in Additional file 1.

### Corn silage

Corn silage from a farm in Neichen (Saxony, Germany) was transported and stored in vacuum-sealed polyester bags at room temperature until its use. Soluble chemicals in the silage were quantified (0.41 ± 0.04 g L^−1^ acetate, 0.21 ± 0.06 g L^−1^ ethanol, and 3.2 ± 0.1 g L^−1^ lactate). The content of VS on a fresh matter basis for the substrate was 27.1 ± 0.1%.

### Source of the adapted community

The syngas-adapted community used as inoculum for the batch cultures originated from two 1.0 L stirred-tank reactors that were being operated near atmospheric pressure (102 kPa) with continuous syngas recirculation (32% H_2_, 32% CO, 16% CO_2_, 2% ethylene, 2% He, and rest N_2_ at 40 mL min^−1^) at a hydraulic retention time of 14 d, pH 6.0, and 32 ℃ [29]. Once a day, 71.4 mL of fermentation broth was harvested and 71.4 mL of fresh mineral medium (Additional file 1: Table S1) containing additionally 12 g L^−1^ acetate and 12 g L^−1^ lactate was fed. Other reactor operation procedures were done as described by Baleeiro et al. [20]. At the time the inoculum was collected, the enrichment reactors had been operated for 83 days and had an average carboxylate composition of 11 g L^−1^ acetate, 3.4 g L^−1^ *n*-butyrate, 2.6 g L^−1^ *n*-caproate, and 1.1 g L^−1^ *i*-butyrate. Methanogenesis rates were negligible (about 2.2 mL CH_4_ L^−1^ d^−1^) in comparison to the syngas consumption rates (150 mL H_2_ L^−1^ d^−1^ and 237 mL CO L^−1^ d^−1^ on average). The original microbial community composition of the adapted community is shown in Additional file 1: Figure S2.

### Analytical methods

The VS content of corn silage was determined according to Strach [30]. To estimate the corn silage degradation during fermentation, a procedure based on the total solids (TS) measurement was applied. At the end of the fermentation, the whole content of each bottle was separated by sieving (0.84 mm mesh size). The retained solids were washed with 50 mL phosphate buffer saline solution (PBS; pH 7.4, 11.8 mM phosphates) for 30 min at room temperature in a rotary shaker at 150 rpm. Afterwards, the resulting mixture was sieved again and the TS content of the solid fraction was determined by drying it at 105 °C until a constant weight was obtained (24–48 h). The difference between the TS content (in %) of the abiotic control and the TS content (in %) of the test bottle was defined as the solids degradation, in percentage points (p.p.).

For measuring pH and concentrations of chemicals in the bottles, 0.3 mL of liquid was collected once a week from each bottle. After measuring pH, liquid samples were centrifuged at 9,000 × g for 10 min and a defined amount of the resulting supernatant was diluted five times with PBS to reach a neutral pH value before being analyzed. For analyzing the concentration of chemicals in corn silage, an elution of 25 g substrate with 250 mL PBS was carried out for 24 h at room temperature [31]. The resulting liquid samples were filtered with 0.22 µm nylon syringe filters before being analyzed via high performance liquid chromatography (HPLC). Concentrations of linear monocarboxylates from formate (C1) to *n*-caprylate (C8), linear alcohols from ethanol (C2) to *n*-hexanol (C6) as well as lactate, *i-*butyrate, *i*-valerate, and *i*-caproate were measured using a 1100 series HPLC-RID/UV system (Agilent Technologies, Germany) equipped with precolumn and column Rezex ROA-Organic Acid H + (8%) (Phenomenex, Germany). For compounds with UV absorption at 280 nm, concentration values obtained via UV were averaged with the values obtained via a refractive index detector (RID). The mobile phase of HPLC was 5 mM H_2_SO_4_ at 0.6 mL min^−1^. A sample injection volume of 20 µL was used and the temperatures of the column oven and RID were kept at 55 ℃ and 50 ℃, respectively. All concentrations of chemicals measured via HPLC in this study can be found in Additional file 2.

The bottle headspace pressure was analyzed using a manometer GDH 14 AN (Greisinger electronic, Germany). Afterwards, 2 mL of gas sample was collected twice a week for gas composition analysis via gas chromatography (GC) with a thermal conductivity detector (TCD). The GC-TCD system measured the fractions of H_2_, CO_2_, CO, ethylene, N_2_, O_2_, and CH_4_ and was described previously by Mohr, et al. [32].

Rates in this study are average rates specific to the volume of broth (50 mL). Average rates in terms of mmols of electron equivalents (e^−^ mmol) were used to compare production and consumption of chemicals under different cultivation conditions. Conversion factors in Additional file 1: Table S2 were used for converting mass values into moles, electron equivalents, and carbon equivalents when necessary. See “Formulae for rate, yield, electron balance, and carbon fixation” in Additional file 1 for detailed information on the calculations. The original experimental dataset including concentration of chemicals, pH, and experimental conditions for each sampling points can be found in the Additional file 2. The calculated yields, carbon fixation rates, production rates of chemicals (in terms of mass, mol, and electron/carbon equivalents), and estimates for biomass consumption via electron balance can be found in Additional file 3. Estimating the biomass consumption via electron balance stands on the assumption that cell biomass and unmonitored chemicals had negligible values in comparison to the whole electron pool. We have previously observed that this assumption holds true for similar reactors with gas and organic substrate co-feeding [20, 33, 34].

### Statistical analysis

To test the significance of certain statements, one-way ANOVA with Tukey’s honest significant differences test was realized with a confidence level of 95% using the R Stats package. In such statements, the p values are given.

### Microbial community analysis

For microbial community analysis, 16S rRNA amplicon sequencing was performed using the Illumina MiSeq platform. Cell pellets were collected from the culture bottles at the end of the experiments. Before being stored at −20 ℃, cell pellets were washed once with an equal volume of PBS (ca. 1.8 mL). DNA was extracted from the cell pellets using the ZymoBIOMICS DNA Miniprep kit (Zymo Research, Germany) with cell disruption within 20 min, following the manufacturer’s instructions for nonsoil samples. DNA quantification and quality assessment, polymerase chain reaction (PCR), and library preparation were done as described by Logroño et al. [35] for 16S rRNA. For PCR, primers for the V3 and V4 regions [36] were used. Filtering, denoising, and taxonomical assignment of the amplicon data were done as described previously [33]. Sequence counts of all samples were rarefied to the read number of the sample with the lowest coverage in the dataset (56,674 counts). Spearman’s correlations (*p* < 0.01) between selected abiotic parameters (production/consumption rates and initial partial pressure of gases) and the relative abundance of the 20 most abundant genera were done using all cultures (*n* = 38).

Raw sequence data for this study was deposited at the European Nucleotide Archive (ENA) under the study accession PRJEB49567 (http://www.ebi.ac.uk/ena/data/view/PRJEB49567).

## Results and discussion

### Comparing different starting conditions

To assess the impact of using a microbial community previously adapted to syngas and lactate (besides the autochthonous corn silage community), we compared inoculated cultures with cultures containing only the corn silage community. Product profiles of the fermentations with syngas (49 kPa CO, 49 kPa H_2_, 24 kPa CO_2_) depending on the way the adapted community was inoculated are presented in Additional file 1: Figure S3. As long as the inoculum did not contain high amounts of carboxylates from the enrichment reactor, i.e., when the inoculation was done with washed cells or with 10 vol % reactor broth, using the adapted community proved to be particularly important for accelerating the consumption of H_2_ and CO. Yet, the maximum *n-*butyrate concentration was 1.2 ± 0.1 g L^−1^ and the adapted community contributed little to produce carboxylates longer than propionate (Additional file 1: Figure S3). Because the addition of reactor broth at the beginning of the fermentations was not beneficial to produce medium-chain carboxylates, only washed cells were used in further tests with the adapted community.

### Effects of CO, H_2_, and syngas

Although inoculating a community adapted to syngas improved syngas consumption and overall carboxylate production (Additional file 1: Figure S3), it was not enough to improve medium-chain carboxylate production. Therefore, we tested the effects of the main syngas components (H_2_ and CO) separately on the fermentation with both the autochthonous and the adapted communities.

**Fig. 2:**
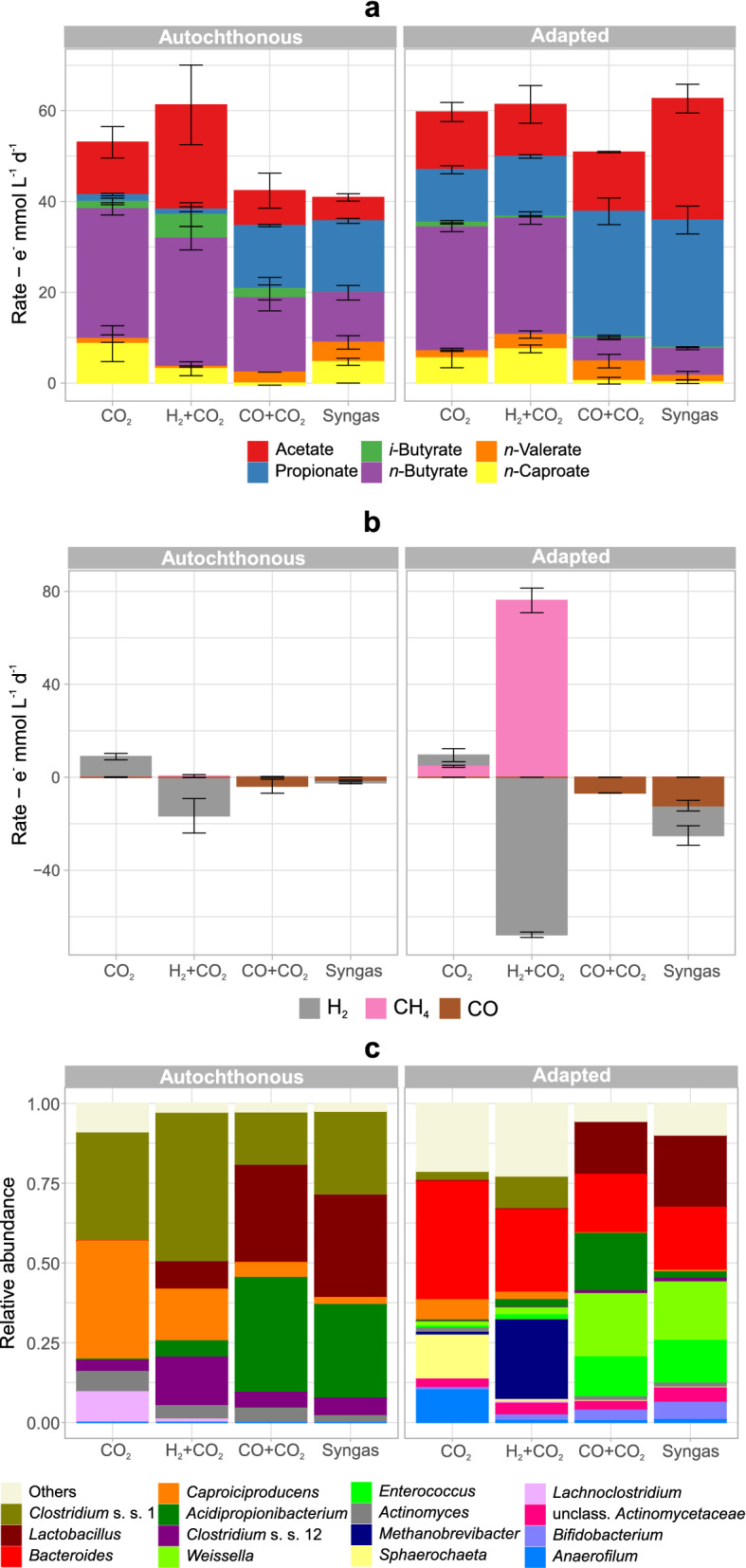
Production and consumption rates for liquid **a** and gaseous **b** chemicals and microbial community composition **c** in fermentations of corn silage with different syngas constituents. The 15 most abundant genera in the set are shown. Mean values of duplicate bottles are shown. Error bars indicate standard errors. Rates are in terms of electron equivalents. S.s.: sensu stricto

Figure 2 shows the production and consumption rates of chemicals (in electron equivalents) together with the community composition under each condition. In cultures with CO, regardless of the community type, propionate was a main electron sink, whereas cultures without CO routed significantly (*p* < 0.001) more electrons to *n*-butyrate (Fig. 2a). The presence of CO (49 kPa) inhibited the production of carboxylates with chains longer than propionate by both communities.

Methane formation occurred only in the culture with the adapted community and was stronger when H_2_ was present and CO was absent (H_2_ + CO_2_, Fig. 2b). In this case, routing of electrons to CH_4_ occurred to a similar extent as H_2_ consumption (76 ± 5 e^−^ mmol CH_4_ L^−1^ d^−1^ and 68 ± 1 e^−^ mmol H_2_ L^−1^ d^−1^), indicating the activity of hydrogenotrophic methanogens in the adapted community. This was confirmed by the high relative abundance of *Methanobrevibacter* under this condition (H_2_ + CO_2_ with the adapted community, Fig. 2c).

The autochthonous community alone consumed some exogenous H_2_ and produced extra acetate when CO was absent (H_2_ + CO_2_, Fig. 2b). Still, the adapted community achieved the highest CO consumption (12 ± 2 e^−^ mmol CO L^−1^ d^−1^), which occurred when both H_2_ and CO were present (*p* = 0.04, Syngas, Fig. 2b). Under this condition, the adapted community presented a net carbon fixation rate of 3.8 ± 0.7 C mmol L^−1^ d^−1^ (equivalent to 167 ± 31 mg CO_2_ L^−1^ d^−1^) (Additional file 1: Figure S4).

At first, H_2_ and CO were assumed to be converted to acetate by autotrophic acetogens such as *Clostridium* sensu stricto 12, a genus that was present (Fig. 2c) and comprises acetogenic species. However, cultures with the adapted community with the highest H_2_ and CO consumptions (excluding methanogenesis) had low relative abundances of *Clostridium* sensu stricto 12. The highest relative abundances were recorded for bacteria expected to be growing heterotrophically on sugars and lactate from the plant biomass: lactic acid bacteria (LAB; *Lactobacillus*, *Weissella*, *Enterococcus*, and *Bifidobacterium*), *Bacteroides*, *Acidipropionibacterium*, *Actinomyces*, and some clostridia (*Clostridium* sensu stricto 1, *Caproiciproducens*, and *Anaerofilum*).

The presence of H_2_ or CO adversely affected *Lachnoclostridium* (autochthonous community) and *Sphaerochaeta* and *Anaerofilum* (adapted community) (Fig. 2c), genera commonly associated with improved lignocellulose degradation [37–40]. Other major shifts in microbial composition were due to the presence of CO. Cultures with CO (49 kPa CO) had greater relative abundances of LAB and *Acidipropionibacterium* at the cost of *Caproiciproducens*, *Methanobrevibacter*, and *Clostridium* sensu stricto 1. Swaps of relative abundances of lactate- and *n*-butyrate-producing bacteria also occurred in other anaerobic systems and depended on pH and organic substrate type [41]. Cultures without inoculation with syngas-adapted cells showed higher relative abundances of *Caproiciproducens, Clostridium* sensu stricto 1 and 12, *Actinomyces*, and *Acidipropionibacterium,* whereas cultures inoculated with the adapted community had higher shares of *Bacteroides*, *Enterococcus*, *Weissella*, and *Bifidobacterium*. *Weissella*, *Bifidobacterium*, and *Enterococcus* were not detected in the adapted community inoculum, indicating that they originated from the corn silage community. *Bacteroides* was present in low abundances in the adapted community inoculum (Additional file 1: Figure S2).

Propionate production by the autochthonous community in the fermentations with CO (Fig. 2a) could be explained by high abundances of *Acidipropionibacterium* (Fig. 2c), which compete with clostridia for lactate [34]. Yet, the adapted community produced even more propionate under the same conditions (Fig. 2a), despite low abundances of *Acidipropionibacterium*. So far, no isolated carboxydotroph is known to convert CO to propionate, but co-cultures of carboxydotrophs and propionate producers have been described [42]. Thus, indirect conversion of CO to propionate intermediated by ethanol is possible. However, high abundances of heterofermentative LAB (i.e., *Weissella* and *Lactobacillus*, which can produce propionate from sugars and lactate [43, 44]) could also explain the observed propionate production.

Interestingly, the relatively high H_2_ and CO consumption by the adapted community in the fermentations with syngas (25.1 e^−^ mmol L^−1^ d^−1^ of gases consumed in comparison to 64.0 e^−^ mmol L^−1^ d^−1^ of carboxylates produced) did not result in much higher carboxylate production than that when only CO_2_ was in the headspace (carboxylate production of 61.6 e^−^ mmol L^−1^ d^−1^) (Fig. 2a). This observation suggests that less plant biomass was consumed when H_2_ or CO were present, which was confirmed both by the analysis of the total solids degradation (Additional file 1: Figure S5a) and by the electron balances (Additional file 1: Figure S5b). According to electron balances, the highest biomass consumption values were 37.5 ± 1.2% e^−^/e^−^ and 37.2 ± 4.7% e^−^/e^−^ and were observed in CO-free cultures that had the capacity to produce CH_4_, i.e., cultures with the adapted community with only CO_2_ or with H_2_ + CO_2_, respectively (Additional file 1: Figure S5b). The presence of H_2_ alone slowed down biomass degradation by the community without methanogenic activity (autochthonous community) from 32.6 ± 9.1% e^−^/e^−^ (CO_2_-only) to 21.3 ± 3.4% e^−^/e^−^ (H_2_ + CO_2_) (Additional file 1: Figure S5b). Still, the slowdown in biomass consumption due to H_2_ presence was overshadowed by the slowdown caused by CO. When CO was present, biomass consumption was between 15.3 ± 5.2% e^−^/e^−^ and 18.7 ± 0.5% e^−^/e^−^ and no clear effect from H_2_ or community type was apparent (Additional file 1: Figure S5b).

Headspaces rich in H_2_ and H_2_ + CO_2_ have already been reported to slow down the degradation of organic waste streams. Arslan, et al. [45] noted a lower hydrolysis rate under a H_2_-rich headspace, but not a change in the final hydrolysis degree of the solid fraction, implying a retarded utilization of the available organic feedstock. There, H_2_ and H_2_ + CO_2_ increased the overall carboxylate production by 47% and 150%, respectively, in comparison to a fermentation with a N_2_ headspace.

Within the same community type (i.e., adapted or autochthonous), community compositions of cultures fed with CO (conditions “Syngas” and “CO + CO_2_”) were very similar. This fact pointed to the importance of CO among all other factors. To get a more detailed view on the effect of CO on the fermentation, further tests with partial pressures lower than 49 kPa CO were carried out.

### Effect of different carbon monoxide concentrations

**Fig. 3:**
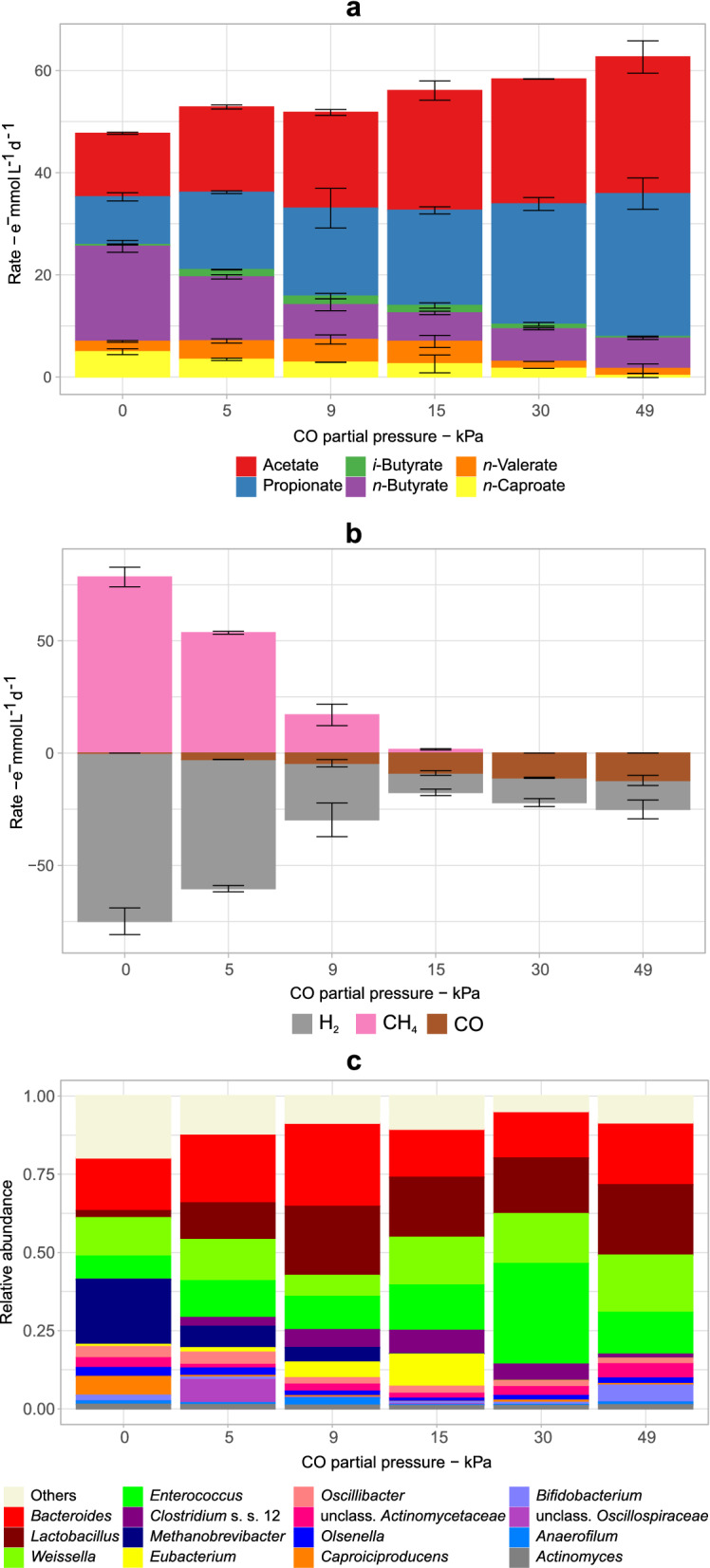
Effect of different partial pressures of CO on the carboxylate spectrum (**a**), gas production and consumption rates (**b**), and on the microbial community at the genus level (**c**). These tests were carried out with bottles inoculated with the adapted community. The 15 most abundant genera in the set are shown. Mean values of duplicate bottles are shown. Error bars indicate standard errors. Rates are in terms of electron equivalents. S.s.: sensu stricto

Production rates for chemicals and community compositions at partial pressures between 0 and 49 kPa CO for cultures with the adapted community are shown in Fig. 3. During this experiment, partial pressures of H_2_ were varied between 98 kPa H_2_ (at 0 kPa CO) and 49 kPa H_2_ (at 49 kPa CO) to maintain a constant availability of electron donors (98 kPa H_2_ + CO) under all conditions (Fig. 1). Additional file 1: Figure S6 shows time profiles of the electron balances under these conditions.

As low as 5 kPa CO was enough to significantly (*p* < 0.001) inhibit the electron flow to *n*-caproate and *n*-butyrate by 32% (Fig. 3a). On the other hand, production of acetate and propionate increased by 115% and 200%, respectively, when CO partial pressure increased from 0 to 49 kPa.

CO partial pressures above 9 kPa did not affect *n*-butyrate production, but inhibited *n*-caproate formation, which dropped to almost zero at 49 kPa CO (Fig. 3a). *n*-Valerate production was only inhibited by more than 15 kPa CO. However, considering that *n*-valerate is produced from propionate [46], this apparent CO tolerance could just be the effect of increased propionate production.

Higher CO partial pressures favored the incorporation of H_2_ and CO into the carboxylate pool (Fig. 3a and b). At 5 and 9 kPa CO, the carboxylate pool increased likely due to partial inhibition of methanogenesis (Fig. 3b) and consequently higher H_2_/CO_2_ availability for acetogens. Nearly complete inhibition of methanogenesis by *Methanobrevibacter* was achieved at about 15 kPa CO, when CH_4_ accounted for less than 3% (1.6 ± 0.3 e^−^ mmol L^−1^ d^−1^) of the total electron sink (61.3 e^−^ mmol L^−1^ d^−1^) (Fig. 3b and c), similar to the study of Esquivel-Elizondo, et al. [47], in which 18 kPa CO completely inhibited methanogens in a mixed culture. From 15 to 30 kPa CO, a visual increase of H_2_ and CO consumption from 8.6 ± 1 to 11 ± 2 e^−^ mmol H_2_ L^−1^ d^−1^ and from 8.9 ± 1.1 to 11.0 ± 0.2 e^−^ mmol CO L^−1^ d^−1^ (Fig. 3b) was observed although it was not statistically significant (*p* > 0.05). H_2_/CO consumptions also remained similar at 30 and 49 kPa CO (Fig. 3b).

Overall, increasing CO partial pressures (Fig. 3c) shaped the community consistently to what was observed previously when 49 kPa CO or 49 kPa H_2_ + 49 kPa CO were used (“Adapted community”, Fig. 2c). LAB, Actinobacteria (e.g., *Acidipropionibacterium* and *Actinomyces*), and *Bacteroides* were either unaffected or profited from increasing CO partial pressures, whereas *Caproiciproducens* rapidly became less abundant under increasing CO partial pressures (Fig. 3c). *Oscillibacter*, which generally had a comparably low abundance, was not inhibited by high CO partial pressures. This is relevant as *Oscillibacter* has previously been associated with *n*-valerate, *n*-caproate, and *n*-caprylate production as well as syngas consumption [48–50] and may be responsible for the formation of carboxylates with longer chains (C ≥ 4) even at high CO pressures. A higher abundance of *Bifidobacterium* (a genus of lactate-producing actinobacteria) was observed at 49 kPa CO. Bacteria from this genus are selectively favored by high propionate concentrations [51], therefore, their higher relative abundance at high CO pressure could be a consequence of high propionate production rates (Fig. 3a) rather than of CO itself.

At high CO partial pressures, the rates of electrons routed to acetate (up to 27 ± 3 e^−^ mmol L^−1^ d^−1^ at 49 kPa CO) were close to the consumption rates of H_2_ and CO (25 e^−^ mmol L^−1^ d^−1^ H_2_ + CO at 49 kPa CO) indicating predominant acetogenic activity. However, the community was majorly composed of Actinobacteria, LAB, and *Bacteroides* whereas the genera known to harbor acetogens (*Clostridium* sensu stricto 12 and *Eubacterium*) were almost absent at this condition (Fig. 3c). To investigate the unexpected absence of acetogens at 49 kPa CO, Spearman correlations analyses were done between rates of chemicals and relative abundances of the community members (Additional file 1: Figure S7). Although consumption of H_2_ and CO_2_ (excluding methanogenesis) correlated significantly (*p* < 0.01) to *Clostridium* sensu stricto 12, consumption of CO only correlated significantly to *Enterococcus* and *Weissella* (Additional file 1: Figure S7).

LAB are not commonly associated to CO consumption. In an exceptional case, Nguyen, et al. [52] reported the isolation of seven LAB (among them, five relatives of *Enterococcus* species) that were able to grow on CO in a medium containing yeast extract. Apart from this, we have not found any other studies characterizing these isolates further. The nickel-containing carbon monoxide dehydrogenase (CODH), known to be involved in anaerobic CO metabolism [53], is not predicted in LAB genomes, and a search in the UniProt database [54] for CODH genes in these bacteria yields entries for the hybrid cluster proteins (HCPs) only (e.g., entries A0A200J077 and A0A5Q2PAN6). HCPs are widely distributed in microorganisms and share homology and catalytic similarities with CODHs [55]. HCPs were pointed out as possible candidates for CO metabolism in organisms that have been proven to consume CO, but lack CODH genes in their genomes, such as *Pyrococcus furiosus* [56] and “Candidatus Galacturonibacter soehngenii” [57], although CODH activity by HCPs has not been observed so far [58].

The link between CO metabolism and the specific Actinobacteria found here is not clear. Here, certain Actinobacteria thrived under high CO partial pressures, but no significant correlations between them and syngas consumption were found (Additional file 1: Figure S7). HCPs are predicted for certain *Acidipropionibacterium*, *Olsenella*, and *Actinomyces* species (e.g., entries A0A3Q9UQ17, A0A0K1F4X5, and A0A2V1KA28 in the UniProt database [54]) and although new classes of Actinobacteria with genes for the bifunctional enzyme CODH/acetyl-CoA synthase (CODH/ACS) were recently shown to possess the Wood−Ljungdahl pathway [59, 60], these acetogenic Actinobacteria are distantly related to the ones enriched here.

### Inhibition of methane production by the combination of ethylene and carbon monoxide

2-Bromoethanosulfonate (2-BES) is the most popular chemical methanogenesis inhibitor in lab-scale fermentations. Yet, this chemical is too expensive to be feasible in commercial-scale anaerobic fermentation [20]. Here, we adopted ethylene as an auxiliary methanogenesis inhibitor during co-fermentation of plant biomass when methanogenic activity was at its peak (at 0 and 5 kPa CO). Conveniently, ethylene is a minor component of syngas produced via biomass gasification [61] and an effective methanogenesis inhibitor at the concentrations in the order of 1% [20].

**Fig. 4:**
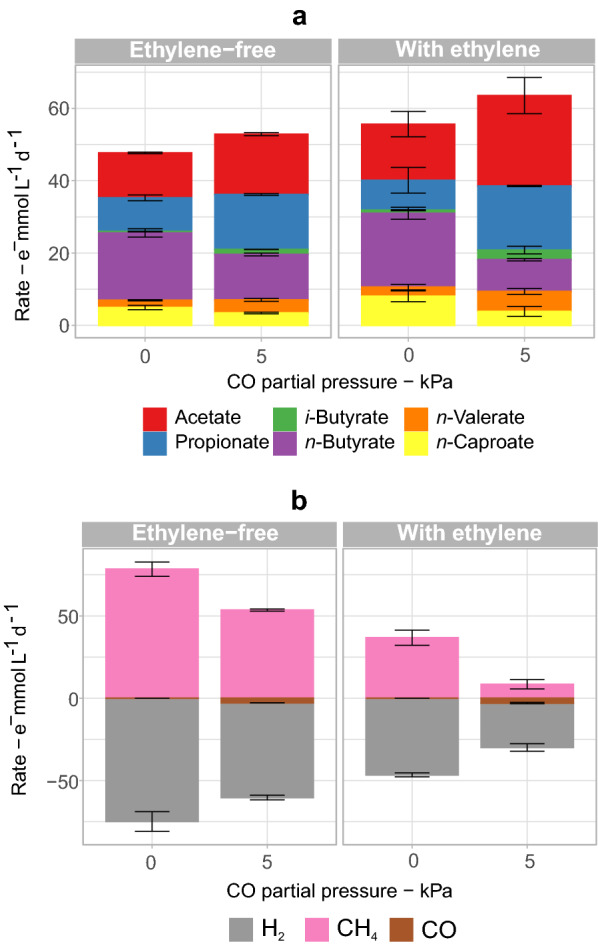
Effect of 1.5 kPa ethylene on the fermentation of corn silage with 0 kPa CO (98 kPa H_2_) and 5 kPa CO (93 kPa H_2_). Production or consumption rates of carboxylates **a** and gases **b** are shown in terms of electron equivalents. Error bars are standard errors. These tests were carried out with bottles inoculated with the adapted community

Overall, cultures with ethylene produced more carboxylates than cultures without ethylene (Fig. 4a). At 0 kPa CO (98 kPa H_2_) and at 5 kPa CO (93 kPa H_2_), ethylene addition increased the carboxylate production by 20% each. However, this increase was mainly due to the additional acetate as ethylene showed no clear effect on the carboxylate chain elongation.

Ethylene alone reduced methane production rates by about half (from 78 ± 4 to 37 ± 5 e^−^ mmol CH_4_ L^−1^ d^−1^), while 5 kPa CO alone reduced methane production rates by about one third (to 102 ± 1 e^−^ mmol CH_4_ L^−1^ d^−1^) (Fig. 4b). When CO and ethylene were applied together, CH_4_ production decreased ninefold (to 8.4 ± 2.8 e^−^ mmol CH_4_ L^−1^ d^−1^). Ethylene had no effect on CO consumption, but increased H_2_ consumption (excluding methanogenesis) from 3.9 ± 2.1 e^−^ mmol H_2_ L^−1^ d^−1^ to 9.8 ± 5.1 e^−^ mmol H_2_ L^−1^ d^−1^ in the case with 5 kPa CO (Additional file 3). Increase in H_2_ consumption due to ethylene presence was observed previously and was attributed to the increased H_2_ availability after inhibiting methanogens [20, 33]. No consumption of ethylene was observed.

**Fig. 5:**
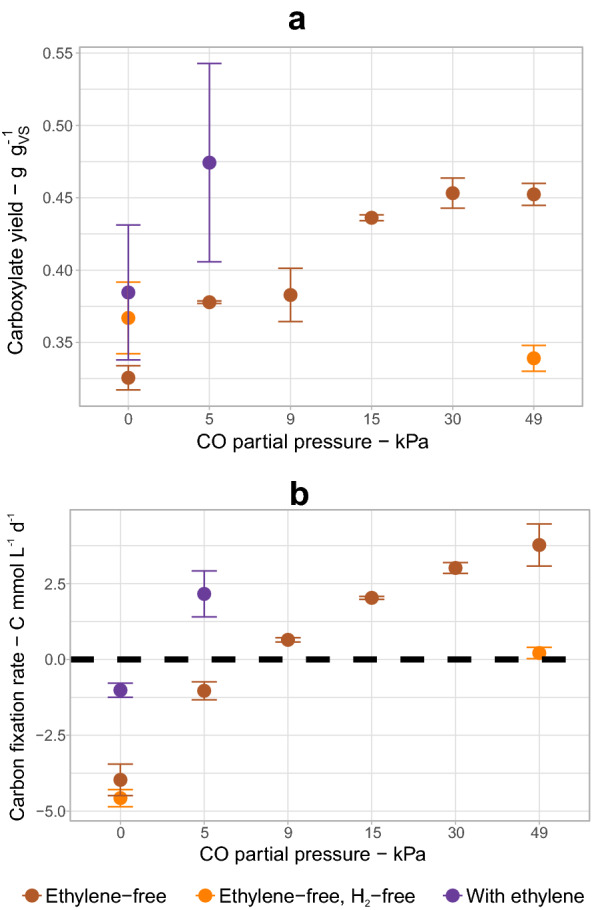
Carboxylate yields from biomass (**a**) and carbon fixation rates (**b**) of fermentations with different gas compositions with CO partial pressures between 0 and 49 kPa. Only results with the adapted community are shown

Figure 5 shows two benchmarks of the fermentation, i.e., carboxylate yields from the plant biomass and carbon fixation rates, at different CO partial pressures. The bottles with ethylene in combination with 5 kPa CO showed with 0.47 ± 0.07 g g_VS_^−1^ the highest carboxylate yield of this study (Fig. 5a). The yield obtained with ethylene + 5 kPa CO was 29% higher than the yield achieved without syngas and 5% higher than the highest yield achieved with syngas containing 30 kPa CO.

The carbon fixation rates followed roughly the same trend as the carboxylate yields. Higher CO partial pressures allowed net carbon fixation up to a maximum of 3.8 ± 0.7 C mmol L^−1^ d^−1^ (equivalent to 167 ± 31 mg CO_2_ L^−1^ d^−1^) (Fig. 5b). This was achieved by inhibiting methanogenesis and by enhancing acetogenic activity, which in turn led to higher carboxylate production (Fig. 4). Cessation of carbon emissions was achieved when at least 9 kPa CO (without ethylene) or 5 kPa CO (with ethylene) was supplied. Fermentation with 5 kPa CO and ethylene was able to fix carbon at a rate of 2.2 ± 0.8 C mmol L^−1^ d^−1^, which was comparable to the carbon fixation rate of 2.03 ± 0.05 C mmol L^−1^ d^−1^ at 15 kPa CO (Fig. 5b).

When used separately, CO and ethylene are imperfect methanogenesis inhibitors. CO is not a selective inhibitor and although ethylene is [20], its inhibitory effect on archaeal hydrogenases can be bypassed by the expression of Fe-only hydrogenases [34]. When used together, small amounts of CO and ethylene had a synergistic effect in inhibiting hydrogenotrophic methanogens. Without methanogens that can misroute electrons from exogenous H_2_, anaerobic fermentation can be turned into a net carbon fixation process.

### The role of formate in carbon monoxide tolerance

**Fig. 6:**
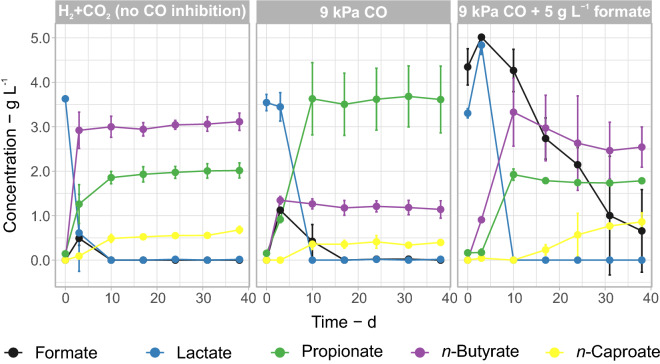
Fermentation profiles of the adapted community under a H_2_ + CO_2_ headspace (uninhibited reference) and at 9 kPa CO with and without added formate. Mean values of duplicate bottles are shown, error bars are standard errors

Under most conditions, transient concentrations of up to 1 g L^−1^ formate were observed (Additional file 2). However, formate profiles were very different in the two pairs of bottles with the autochthonous community in the presence of syngas (Fig. 1). In one pair of bottles (Autochthonous community with syngas, Fig. 2), formate accumulated in the first 10 days and then remained stable at about 2.14 ± 0.06 g L^−1^ (high formate, Additional file 1: Figure S8). In this case, *n*-butyrate and *n*-caproate concentrations were clearly higher (1.7 ± 0.2 and 0.66 ± 0.09 g L^−1^, respectively) in comparison to a pair of bottles with low formate concentrations under the same conditions (0.5 ± 0.6 and 0.3 ± 0.1 g L^−1^, respectively) (low formate, Additional file 1: Figure S8). From this observation, we suspected a relationship between formate and fermentative bacteria overcoming CO inhibition, similar to what has been observed in pure cultures of *A. woodii* [28]. We tested this hypothesis by adding 5 g L^−1^ formate at the beginning of the fermentation at 9 kPa CO. With 5 g L^−1^ formate, the production of *n*-butyrate and *n*-caproate at 9 kPa CO (3.3 ± 0.8 g L^−1^ and 0.9 ± 0.2 g L^−1^, respectively) was similar to that of the fermentations uninhibited by CO (3.1 ± 0.2 and 0.68 ± 0.08 g L^−1^, respectively) (Fig. 6). Carbon balances indicate that the consumed formate (equivalent to 2.1 ± 0.3 C mmol L^−1^ d^−1^) was likely converted to CO_2_. After discounting CO_2_ routed to methane, the bottle pair with added formate produced 4.3 ± 1.5 mmol CO_2_ L^−1^ d^−1^, in comparison to only 1.6 ± 0.9 mmol CO_2_ L^−1^ d^−1^ from the bottles with no formate addition (Additional file 3).

**Fig. 7:**
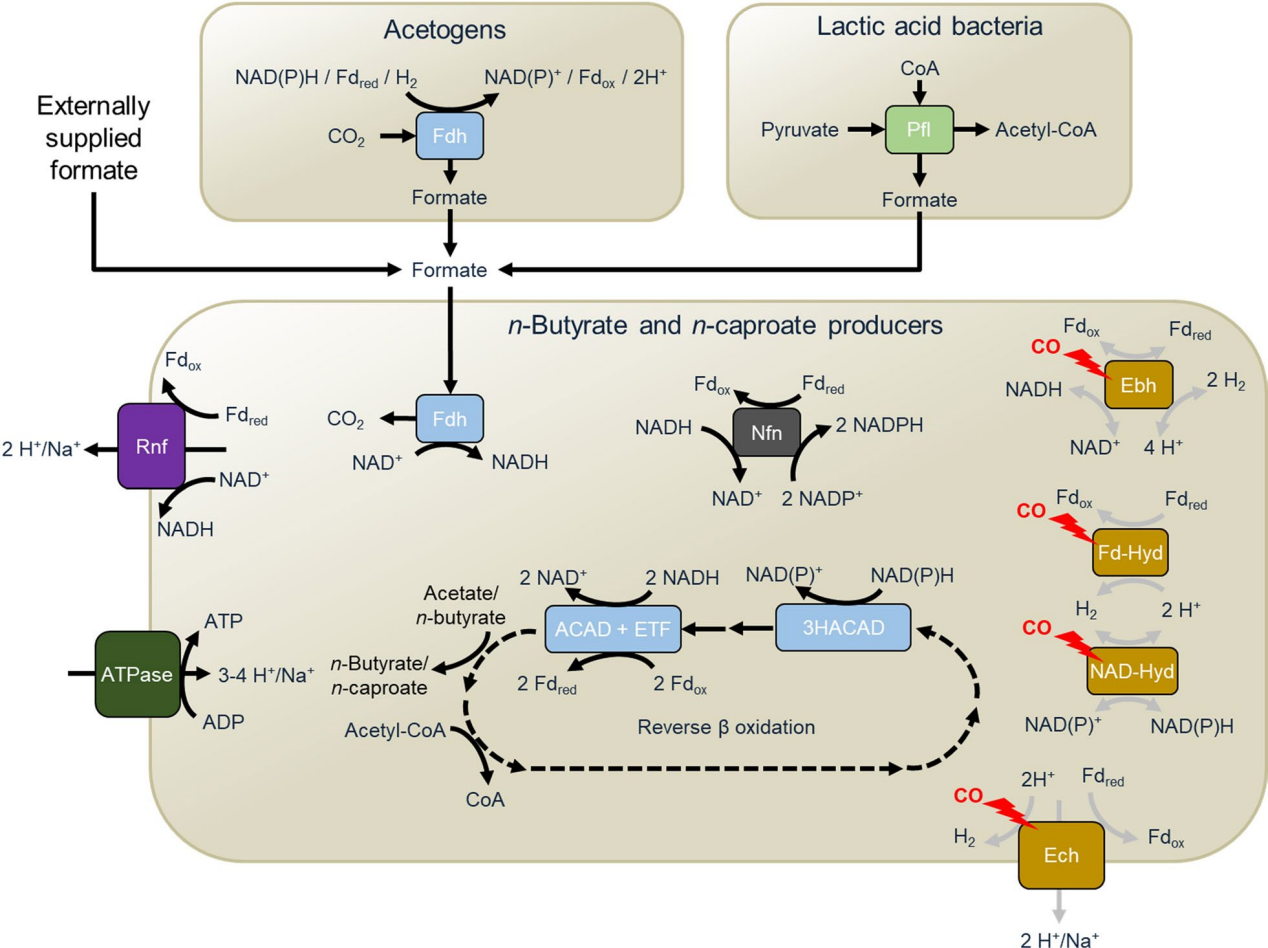
Theoretical model of formate-induced CO tolerance in *n*-butyrate and *n*-caproate producers performing reverse β-oxidation. The CO-inhibited enzymes in the lower box are typically involved in energy conservation in anaerobic acidogenic bacteria that lack cytochromes. Chain-elongating bacteria can compensate for this inhibition by oxidizing formate, thereby providing reduction equivalents needed for reverse β-oxidation. Dashed arrows represent sequential metabolic steps and gray arrows represent CO-inhibited processes. *3-HACAD* 3-hydroxy-acyl-CoA dehydrogenase, *ACAD* acyl-CoA dehydrogenase, *Ebh* electron bifurcation hydrogenase, *Ech* energy-conserving hydrogenase, *ETF* electron-transferring flavoproteins A and B, *Fdh* formate dehydrogenase, *Fd-Hyd* Fd-dependent hydrogenase, *NAD-Hyd* NAD-dependent hydrogenase, *Nfn* NAD(P)^+^ transhydrogenase, *Pfl* pyruvate formate lyase

Based on the observations in Fig. 6 and Additional file 1: Figure S8, we propose a theoretical model to explain how formate could help *n*-butyrate- and *n*-caproate-producing bacteria to overcome inhibition by CO (Fig. 7). These bacteria depend on hydrogenases to re-oxidize Fd_red_ (coupling it with H_2_ formation) or to regenerate their NAD(P)H pools. NAD(P)H is required for the elongation cycles with the acyl-CoA and 3-hydroxy-acyl-CoA dehydrogenases (ACAD and 3-HACAD, respectively). On the other hand, formate dehydrogenases (Fdh) can be found in many acidogenic bacteria and these enzymes are assumed to be less sensitive to CO than hydrogenases [62]. Thus, *n*-butyrate and *n*-caproate producers that have an Fdh can couple formate oxidation with NAD^+^ reduction and bridge the gap left by hydrogenases inhibited by CO.

Formate is an extracellular electron carrier between anaerobic microorganisms such as methanogens, acetogens, and sulfate-reducing bacteria [63, 64]. Under the conditions used here, we expect endogenous formate production to occur mainly in two different ways (Fig. 7): (i) during the conversion of pyruvate to acetyl-CoA via pyruvate formate lyase (Pfl) often found in LAB [44] and (ii) as an intermediate in the metabolism of acetogens after the fixation of one CO_2_ molecule with an electron pair (in the form of H_2_, NADH, or Fd^−2^) via Fdh in the methyl branch of the Wood−Ljungdahl pathway [65, 66]. According to the UniProt database [54], Fdh is predicted from the genomes of *C. butyricum* (*Clostridium* sensu stricto 1), *Megasphaera elsdenii*, *Eubacterium limosum*, and *C. luticellarii* (*Clostridium* sensu stricto 12). However, some other well-known acidogens such as *C. kluyveri* and *C. tyrobutyricum* (both belonging to *Clostridium* sensu stricto 12) do not have annotated genes for Fdh.

Additional file 1: Figure S9 presents the community compositions at the genus level for the cultures with inhibition or tolerance to CO. Higher relative abundances of *Clostridium* sensu stricto 1 were observed in the fermentation with the autochthonous community under the conditions CO + formate and H_2_ + CO_2_ (no CO inhibition). In the adapted community, the presence of formate caused the absence of *Bacteroides,* which in contrast accounted for about 25% of the bacteria in the bottles without formate, and increased the share of less abundant genera (grouped in “others”), in similarity to uninhibited bottles. 16S rRNA amplicon sequencing does not provide direct information on the presence or absence of the Fdh gene in bacteria and we were not able to investigate links between Fdh-containing bacteria and formate-induced CO tolerance. Therefore, the proposed metabolic mechanism in Fig. 7 should be further tested experimentally. Preferably, this should be done in less complex systems such as co-cultures or single cultures, while monitoring the expression of the respective genes.

Besides, there are other conceivable explanations for how formate restores the production of *n*-butyrate and *n*-caproate in CO-inhibited cultures. It is possible that formate favors *n*-butyrate and *n*-caproate producers by selectively inhibiting propionate producers that compete for lactate and sugars or that acetogens can produce more chain elongation substrates (i.e., ethanol and acetate) under the presence of formate. Still, community analysis does not corroborate these theories. Propionate producers (e.g., *Acidipropionibacterium*) did not become less abundant and acetogens (e.g., *Clostridium* sensu stricto 12 and *Eubacterium*) did not become more abundant under the presence of formate (Additional file 1: Figure S9). Direct conversion of formate to *n*-butyrate could be another explanation. However, production of *n*-butyrate from formate at levels relevant to a bioprocess is unexpected since this conversion is energetically unfavorable [67]. To the best of our knowledge, pure cultures of acetogenic bacteria produce at best trace amounts of *n*-butyrate (and *n*-caproate) from formate. For instance, *E. limosum* can grow on formate but without *n*-butyrate production [68]. It is also possible that formate upregulates *n*-butyrate production pathways that are not necessarily coupled to growth. Future experiments with controls accounting for the isolated effect of formate (i.e., added formate, without CO) could shed light on this possibility. Even though *C. kluyveri* does not possess annotated genes for Fdh, adding formate to the well-studied *C. kluyveri* + acetogen co-cultures could prove useful to test other conceivable explanations for the observed effect of formate on production of elongated carboxylates.

## Conclusion

CO toxicity and inhibition of methanogens were the most important factors influencing the carboxylate production. Increasing CO partial pressures completely reshaped the microbial community and shifted the product spectrum from C ≥ 4 carboxylates and methane to acetate and propionate. H_2_ in the headspace had limited effects. When H_2_ was present, it favored the growth of hydrogenotrophic methanogens, inhibited some bacterial genera commonly associated with lignocellulose degradation, and retarded biomass decomposition. From a sustainability perspective, a syngas composition with low partial pressures of CO and ethylene and high partial pressure of H_2_ was particularly interesting since it showed a synergistic effect in inhibiting hydrogenotrophic methanogens and achieving net carbon fixation via acetogenesis. This syngas mixture yielded 29% more carboxylates than a conventional fermentation (with a N_2_/CO_2_ headspace) despite slower biomass degradation rates, hence reducing the dependence of anaerobic fermentation on biomass availability. Nevertheless, a big share of this improvement was due to increased production of acetate, an ordinary syngas fermentation product, and propionate. To achieve higher yields of long-chain carboxylates (C ≥ 4), we recommend testing the concept by operating the fermentation as a continuous process. In this way, hydrolytic and chain-elongating bacteria that cope well with both CO and complex feedstocks could be selected.

A surprising absence of acetogenic clostridia under the conditions with the highest H_2_ and CO consumption rates hints that there is still much to learn about the community dynamics behind syngas + biomass systems. This observation led us to speculate if mixotrophic actinobacteria or mixotrophic LAB were the ones routing electrons from H_2_/CO into acetate and propionate. We could not draw any conclusions on this possibility here and future attempts to isolate and characterize these possible mixotrophs are recommended.

As little as 5 kPa CO was sufficient to hinder *n*-caproate and *n*-butyrate production. However, this inhibition was not observed in cultures with CO in which formate concentration remained above 2 g L^−1^. To the best of our knowledge, formate-induced tolerance to CO has not yet been reported, neither for mixed cultures nor for *n*-butyrate- and *n*-caproate-producing pure cultures. We postulate that formate could be consumed by fermentative bacteria to maintain their NAD(P)H pool via formate dehydrogenase, thus bridging the gap left by hydrogenases inhibited by CO. Further experiments are needed to test this hypothesis. If true, this feature could be exploited in designing bioelectrochemical systems with CO or in fermentation technologies based on the C1 substrates.

## Supplementary Information

**Additional file 1. **Mineral medium preparation, Further details on the experimental setup, Formulae for rate, yield, electron balance, and carbon fixations, and Inoculation with a syngas-adapted community. **Table S1.** Mineral medium composition. **Table S2.** Conversion factors. **Figure S1.** Concentration of organic acids and electron balances in the abiotic controls. **Figure S2.** Composition of the syngas-adapted community used as inoculum. **Figure S3.** Concentration of organic acids and electron balances during the fermentation of corn silage + syngas (H_2_/CO/CO_2_ ratio of 49:49:24 kPa) depending on the inoculation with the syngas-adapted community. **Figure S4.** Carbon fixation rates achieved with the autochthonous corn silage community and with the inoculated (adapted) community at different components of syngas. **Figure S5.** Estimates for the consumption of corn silage under different headspaces and with different inocula. **Figure S6.** Electron balance kinetics for fermentations of corn silage in the presence of different partial pressures of CO. **Figure S7.** Spearman correlations between selected abiotic parameters and the relative abundances of the 20 most abundant genera in the dataset (n = 38, p < 0.01). **Figure S8.** Fermentation profiles of the autochthonous community under a H_2_ + CO_2_ headspace (uninhibited reference) and under a syngas headspace (49 kPa CO) with low and high formate accumulation. **Figure S9.** Composition of the autochthonous community and the syngas-adapted community depending on CO inhibition and formate availability.

**Additional file 2. **Original experimental data including concentration of all chemicals and detailed information for each condition.

**Additional file 3. **Calculated rates in mass, molar, and electron/carbon equivalents for each pair of replicates. Final concentration of chemicals, carbon fixation rates, and carboxylate yields for each pair of replicates. Estimation of the consumed biomass via electron balance.

## Data Availability

The raw sequence reads generated during the current study are available in the European Nucleotide Archive (ENA) under the accession PRJEB49567 (http://www.ebi.ac.uk/ena/data/view/PRJEB49567).
