# Supplementary material for: Formate-induced CO tolerance and methanogenesis inhibition in fermentation of syngas and plant biomass for carboxylate production
Source: Biotechnol Biofuels Bioprod. 2023 Feb 17;16:26. doi: 10.1186/s13068-023-02271-w (PMC9936662; doi:10.1186/s13068-023-02271-w)
Supplement: Supplementary file 1 — Additional file 1. Mineral medium preparation, Further details on the experimental setup, Formulae for rate, yield, electron balance, and carbon fixations, and Inoculation with a syngas-adapted community. Table S1. Mineral medium composition. Table S2. Conversion factors. Figure S1. Concentration of organic acids and electron balances in the abiotic controls. Figure S2. Composition of the syngas-adapted community used as inoculum. Figure S3. Concentration of organic acids and electron balances during the fermentation of corn silage + syngas (H2/CO/CO2 ratio of 49:49:24 kPa) depending on the inoculation with the syngas-adapted community. Figure S4. Carbon fixation rates achieved with the autochthonous corn silage community and with the inoculated (adapted) community at different components of syngas. Figure S5. Estimates for the consumption of corn silage under different headspaces and with different inocula. Figure S6. Electron balance kinetics for fermentations of corn silage in the presence of different partial pressures of CO. Figure S7. Spearman correlations between selected abiotic parameters and the relative abundances of the 20 most abundant genera in the dataset (n = 38, p < 0.01). Figure S8. Fermentation profiles of the autochthonous community under a H2 + CO2 headspace (uninhibited reference) and under a syngas headspace (49 kPa CO) with low and high formate accumulation. Figure S9. Composition of the autochthonous community and the syngas-adapted community depending on CO inhibition and formate availability. [file 13068_2023_2271_MOESM1_ESM.pdf]

Additional file 1

Formate-induced CO tolerance and methanogenesis inhibition in fermentation of syngas and plant biomass for carboxylate production

Flávio C. F. Baleeiro, Lukas Varchmin, Sabine Kleinsteuber, Heike Sträuber, Anke Neumann

MINERAL MEDIUM PREPARATION. 2
FURTHER DETAILS ON THE EXPERIMENTAL SETUP. 2
FORMULAE FOR RATE, YIELD, ELECTRON BALANCE, AND CARBON FIXATION. 2
Table S1. 4
Table S2. 5
Figure S1. 5
Figure S2. 6
Figure S3. 7
INOCULATION WITH A SYNGAS-ADAPTED COMMUNITY 7
Figure S4. 8
Figure S5. 9
Figure S6. 10
Figure S7. 11
Figure S8. 12
Figure S9. 13

MINERAL MEDIUM PREPARATION. All medium ingredients (Table S1), except vitamins and cysteine, were mixed under oxic conditions. After adjusting the pH to 6.0 with 4 M NaOH, the mixture was stirred (700 rpm) in an anaerobic chamber (1 – 5% H_2 with the rest as N_2) for at least 3 hours to be make it anoxic. The anoxic solution was transferred to a glass bottle and capped and autoclaved at 121°C for 20 minutes. Stock solutions with vitamins and with cysteine were prepared separately and were only mixed with the rest of the ingredients on the day of the start of the batch experiment inside the anaerobic chamber. The vitamin and cysteine stock solutions were stored in serum bottles sealed with butyl rubber stoppers and aluminum crimps. The concentrated 30 g L^{-1} cysteine hydrochloride solution was prepared with anoxic, deionized water inside an anaerobic chamber and was made sterile by autoclaving at 121°C for 20 minutes. The 1000-fold concentrated vitamin stock solution was sterilized with a nylon 0.22 µm syringe filter and then made anoxic with at least 15 pressurization-depressurization cycles with pure N_2 lasting 2 min each.

FURTHER DETAILS ON THE EXPERIMENTAL SETUP. The experiments described in Fig. 1 were divided into three batches lasting between 31 and 38 d depending on the carboxylate production. The first batch lasted 31 d and compared different starting communities (the autochthonous and the syngas-adapted communities) as well as the way of inoculating the adapted community (with washed cells, 10 vol % reactor broth, or 100 vol % reactor broth). This batch was realized under a syngas atmosphere with 49 kPa H_2, 49 kPa CO, and 24 kPa CO_2. The second batch lasted 35 d and compared the effects of the presence or absence of H_2 and CO on the autochthonous community and on the adapted community. The third batch lasted 38 d and tested the adapted community under intermediate partial pressures of CO (from 0 to 30 kPa). During this batch, the effect of 1.5 kPa ethylene (at 0 and 5 kPa CO) and the effect of the addition of 5 g L^{-1} formate (at 9 kPa CO) were also tested.

FORMULAE FOR RATE, YIELD, ELECTRON BALANCE, AND CARBON FIXATION. Specific average rates of chemical i (in terms of mg L^{-1} d^{-1}, mmol L^{-1} d^{-1}, e^- mmol L^{-1} d^{-1}, or C mmol L^{-1} d^{-1}) were calculated as described in Equation 1.

$$r_i = \frac{n_{f,i} - n_{0,i}}{V_{broth} \times t} \quad (1)$$

Where $n_{f,i}$ and $n_{0,i}$ are the amounts of chemical i at the final and beginning of the batch, respectively, in terms of mg, mmol, e^- mmol, or C mmol; V_{broth} is the initial volume of broth in L; and t is the batch duration in d. As a convention, production rates are positive and consumption rates are negative.

Carboxylate yields from biomass (in g g_{VS}^{-1}) were calculated considering all monitored carboxylates except lactate and is described in Equation 2.

$$Y = \frac{\sum_j (C_{f,j} - C_{0,j})}{C_{0,VS}} \times V_{broth} \quad (2)$$

Where $C_{f,j}$ and $C_{0,j}$ are the concentrations of carboxylate j (excluding lactate) at the final and beginning of the batch, respectively, in g L^{-1}; $C_{0,VS}$ is the initial concentration of volatile solids, in g_{VS} L^{-1}.

To illustrate kinetic profiles of electron balances, the amounts of all chemicals were set to zero at the beginning of the batch. Chemicals in the liquid phase follow Equation 3.

$$e'_{k,i} = e_{k,i} - e_{0,i} \quad (3)$$

Where $e'_{k,i}$ is the produced (+) or consumed (−) amount of chemical *i* at sampling point *k*, in e^- mmol; $e_{k,i}$ is the total amount of chemical *i* in the system at sampling point *k*, in e^- mmol; and $e_{0,i}$ is the total amount of chemical *i* in the system at the beginning of the batch, in e^- mmol.

To keep track of the balances in the gas phase, the cumulative production (+) or consumption (−) of the gas had to be used to account for the headspace purges/replenishments of bottles. If *k* is a sampling point in which no gas replenishment was done, Equation 4 was used.

$$n'_{k,g} = {y_{k,g} P_k V_{k,hs}}/{RT_k} - {y_{k-1,g} P_{k-1} V_{k-1,hs}}/{RT_{k-1}} + n'_{k-1,g} \quad (4)$$

Where $n'_{k,g}$ and $n'_{k-1,g}$ are the cumulative production (+) or consumption (−) of a gas *g* at sampling points *k* and *k-1*, in mmol; $y_{k,g}$ and $y_{k-1,g}$ are the molar fractions of gas *g* at sampling points *k* and *k-1*, respectively; P_k and P_{k-1} are the absolute pressures at sampling points *k* and *k-1*, respectively, in Pa; $V_{k,hs}$ and $V_{k-1,hs}$ are the headspace volumes at sampling points *k* and *k-1*, respectively, in L; T_k and T_{k-1} are the absolute temperatures at sampling points *k* and *k-1*, respectively, in K; and *R* is the gas constant, 8.3145 Pa L K^{-1} $mmol^{-1}$.

If *k* is a sampling point in which a gas replenishment was done, cumulative gas production was calculated according to Equation 5.

$$n'_{k,g} = n'_{k-1,g} \quad or \quad n'_{0,g} = 0 \quad (5)$$

The rate of carbon fixation (in C mmol L^{-1} d^{-1}) was calculated as described in Equation 5.

$$r_{C,fixed} = -(r_{C,CH4} + r_{C,CO2} + r_{C,CO}) \quad (6)$$

where $r_{C,CH4}$, $r_{C,CO2}$, and $r_{C,CO}$ are the production (+) or consumption (−) rates of CH_4, CO_2, and CO, respectively, in carbon equivalents.

Table S1. Mineral medium composition.

Concentration of major components - g L^{-1}	
NH_4Cl	1.61
KH_2PO_4	13.6
NaCl	2.0
NaOH (approx.)	>1.0 (correction to pH 6.0)
Concentration of minor components - mg L^{-1}	
$MgCl_2 \times 6\ H_2O$	54
$CaCl_2 \times 2\ H_2O$	65
Resazurin	0.5
$FeCl_2 \times 4\ H_2O$	1.5
Cysteine hydrochloride	30
Concentration of trace elements - µg L^{-1}	
$CuCl_2 \times 2\ H_2O$	2.0
$CoCl_2 \times 6\ H_2O$	190
$MnCl_2$	100
$Na_2MoO_4 \times 2\ H_2O$	36
$NiCl_2 \times 6\ H_2O$	24
$Na_2WO_4 \times 2\ H_2O$	20
$Na_2SeO_3 \times 5\ H_2O$	3.0
$ZnCl_2$	70
H_3BO_3	6.0
Concentration of vitamins - µg L^{-1}	
Biotin	20
Folic acid	20
Pyridoxine	100
Thiamine	50
Riboflavin	50
Niacin	50
Calcium pantothenate	50
Cobalamin	20
p-Aminobenzoic acid	80
Lipoic acid	50

Table S2. Conversion factors.

Compound (formula)	Molar mass (g mol^{-1})	mol C mol^{-1}	mol e$^-$ mol^{-1}
Biomass ($C_1H_{1.8}O_{0.5}N_{0.2}$)	24.6	1.0	4.2
Formic acid/formate ($C_1H_2O_2$)	46.0	1.0	2.0
Acetic acid/acetate ($C_2H_4O_2$)	60.0	2.0	8.0
Ethanol (C_2H_6O)	46.0	2.0	12.0
Propionic acid/propionate ($C_3H_6O_2$)	74.0	3.0	14.0
Lactic acid/lactate ($C_3H_6O_3$)	90.0	3.0	12.0
n-Butyric acid/*n*-butyrate ($C_4H_8O_2$)	88.0	4.0	20.0
i-Butyric acid/*i*-butyrate ($C_4H_8O_2$)	88.0	4.0	20.0
n-Valeric acid/ *n*-valerate ($C_5H_{10}O_2$)	102.1	5.0	26.0
n-Caproic acid/ *n*-caproate ($C_6H_{12}O_2$)	116.1	6.0	32.0
H_2	2.0	0.0	2.0
CO_2	44.0	1.0	0.0
CO	28.01	1.0	2.0
CH_4	16.0	1.0	8.0

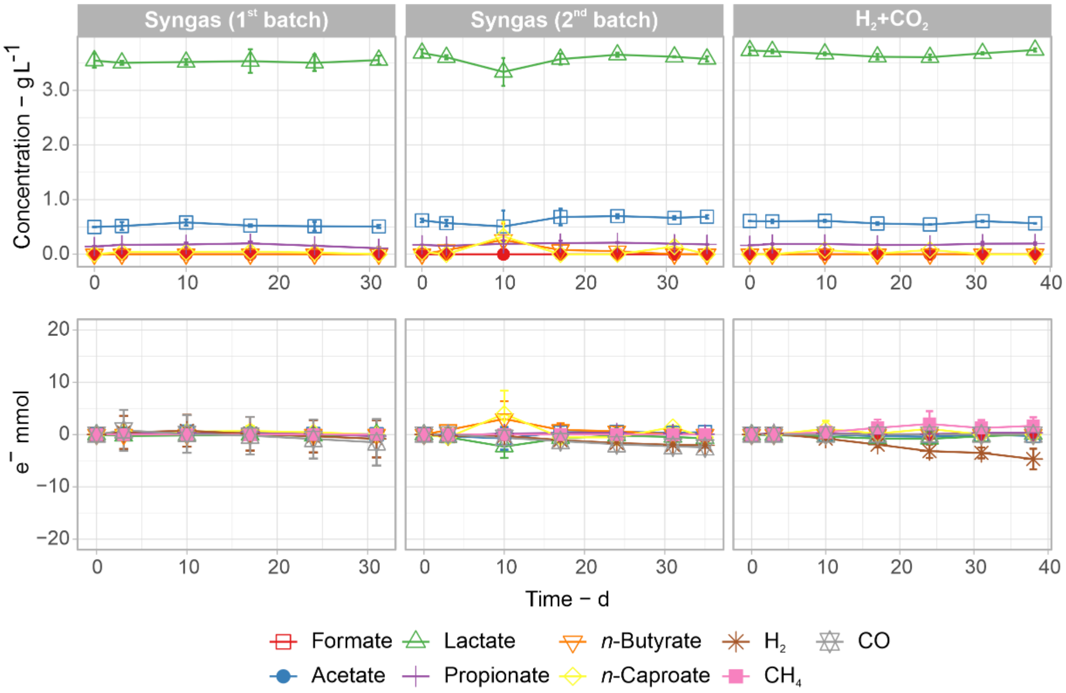

Figure S1. Concentration of organic acids and electron balances in the abiotic controls. Mean values of duplicate bottles are shown. Error bars indicate standard errors.

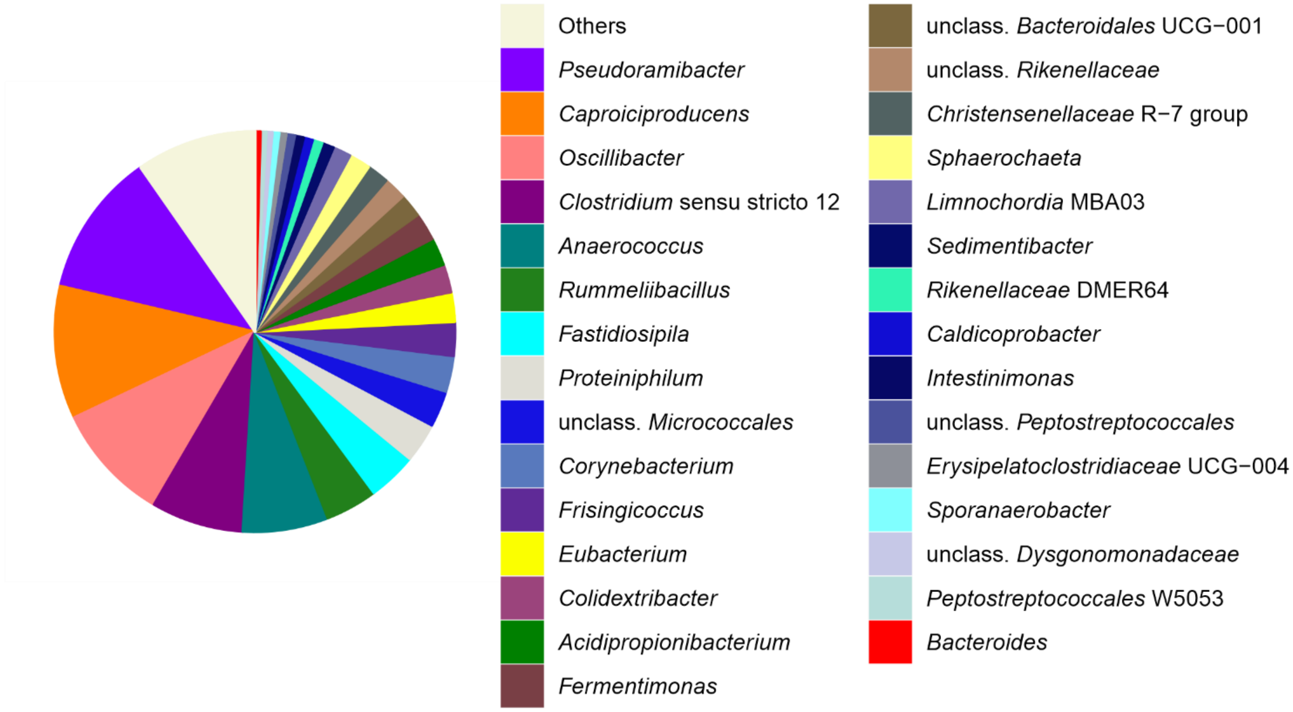

Figure S2. Composition of the syngas-adapted community used as inoculum. The 30 most abundant genera are shown.

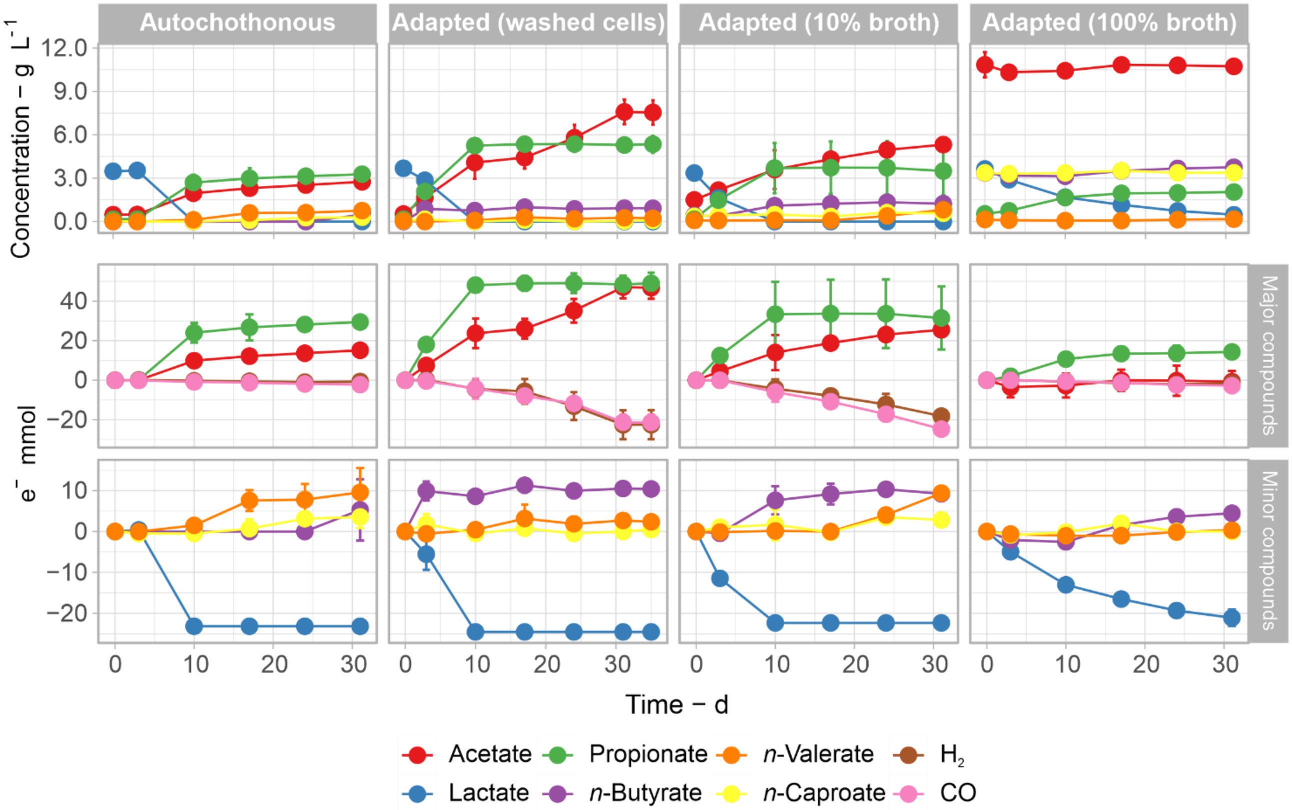

Figure S3. Concentration of organic acids and electron balances during the fermentation of corn silage + syngas ($H_2/CO/CO_2$ ratio of 49:49:24 kPa) depending on the inoculation with the syngas-adapted community (see Figure S2). From left to right: autochthonous community (corn silage community only), inoculation with the adapted community (washed cells), inoculation with 10 vol% reactor broth, and inoculation with 100 vol% reactor broth. Mean values of duplicate bottles are shown. Error bars indicate standard errors.

INOCULATION WITH A SYNGAS-ADAPTED COMMUNITY. Regardless of the initial microbial community, production of carboxylates with more than three carbon atoms (i.e. *n*-butyrate, *n*-valerate, and *n*-caproate) remained less than 1.5 g L^{-1} (Figure S3). Besides, no methane formation was observed.

Cultures that contained only the autochthonous community of corn silage presented a longer lag phase than bottles inoculated with the adapted community, as evidenced by the late start of lactate consumption. Still, the autochthonous community started to slowly consume CO in the middle of the batch.

In comparison to the fermentations with the corn silage community alone, inoculation with the adapted community via washed cells or via the addition of 10 vol% reactor broth caused higher production of acetate and propionate. In addition, the highest H_2 and CO consumption was also observed in these two sets.

Originally, the reactor broth used for the inoculation contained a high concentration of carboxylates (ca. 11 g L^{-1} acetate, 4.5 g L^{-1} C4 carboxylates, and 2.6 g L^{-1} *n*-caproate). Therefore, when 100 vol% of the enrichment reactor broth was used as inoculum, the carboxylates present in the broth strongly inhibited the fermentation of corn silage and syngas, and only a slow conversion of the lactate from corn silage into propionate was observed. The carboxylates from the broth that entered the bottles via inoculation were neither consumed nor converted into alcohols.

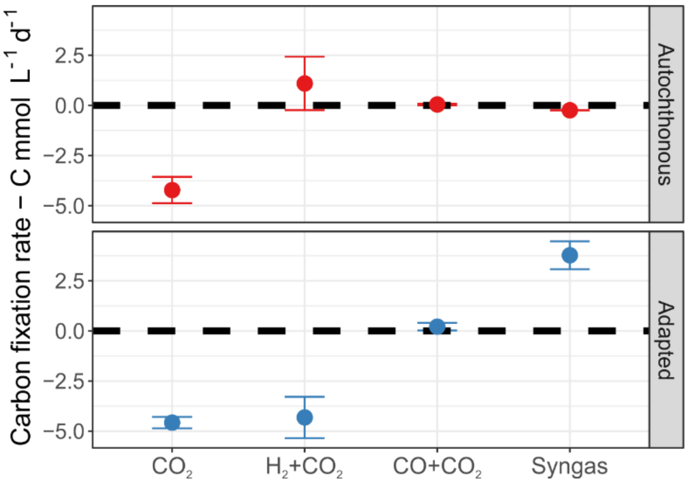

Figure S4. Carbon fixation rates achieved with the autochthonous corn silage community and with the inoculated (adapted) community at different components of syngas. Mean values of duplicate bottles are shown. Error bars indicate standard errors.

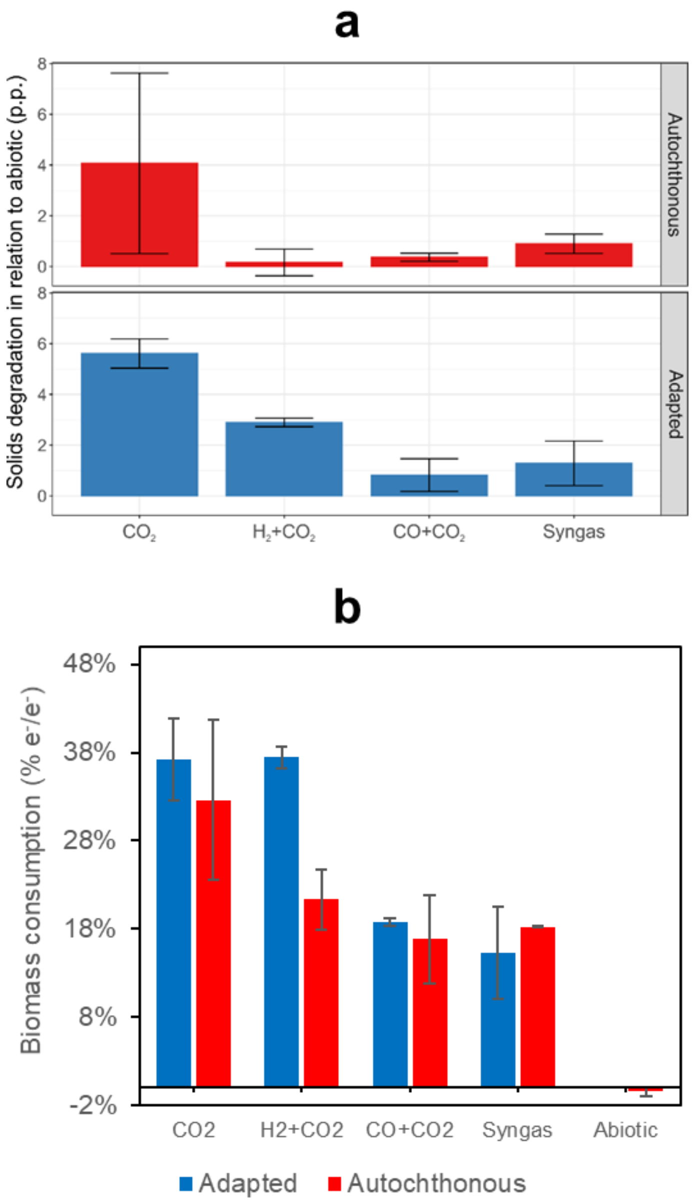

Figure S5. Estimates for the consumption of corn silage under different headspaces and with different inocula. **a** Using the method to quantify solids degradation relative to the abiotic controls (in percentage points, p.p.) and **b** using electron balances (in % of electron equivalents). Mean values of duplicate bottles are shown. Error bars indicate standard errors.

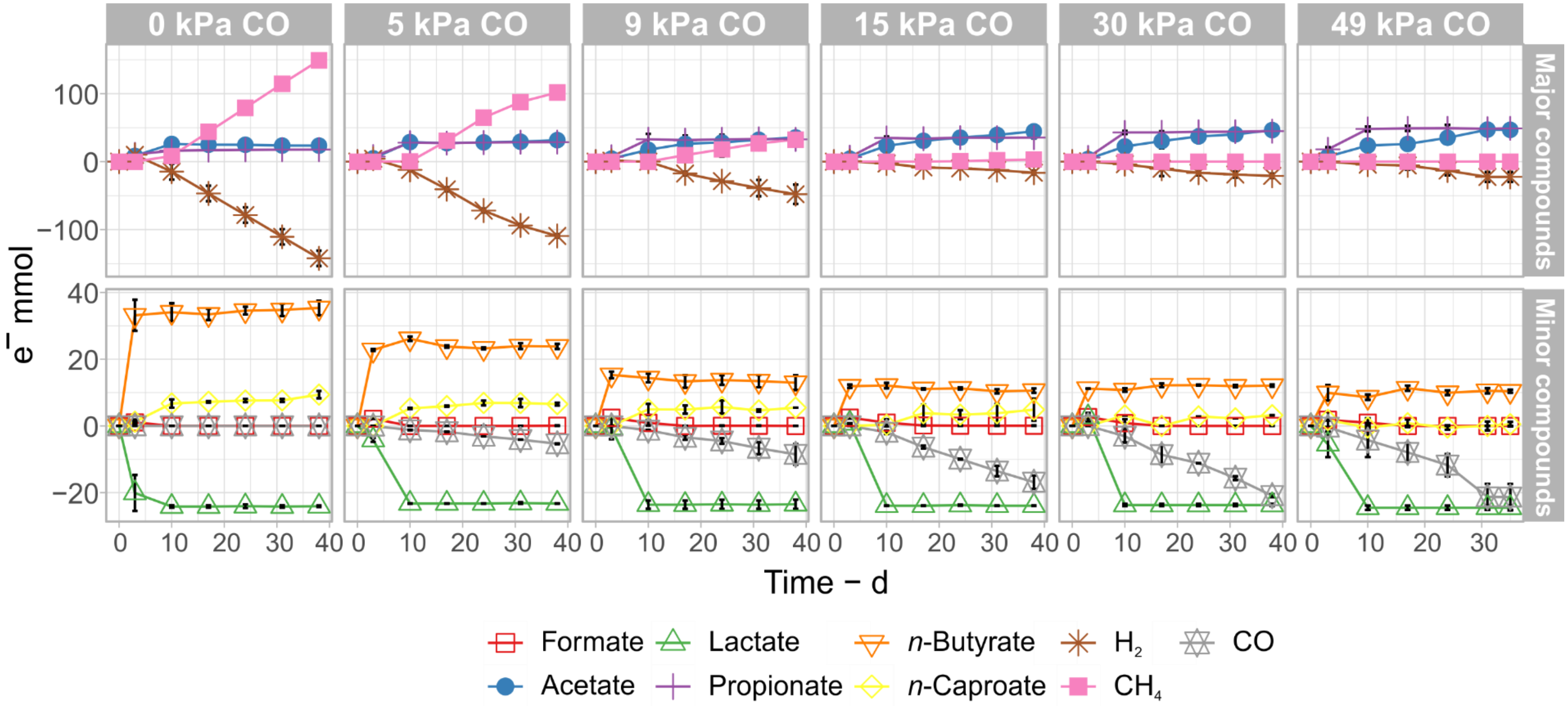

Figure S6. Electron balance kinetics for fermentations of corn silage in the presence of different partial pressures of CO. Cultures inoculated with the adapted community were used for this experiment. Mean values of duplicate bottles are shown. Error bars indicate standard errors.

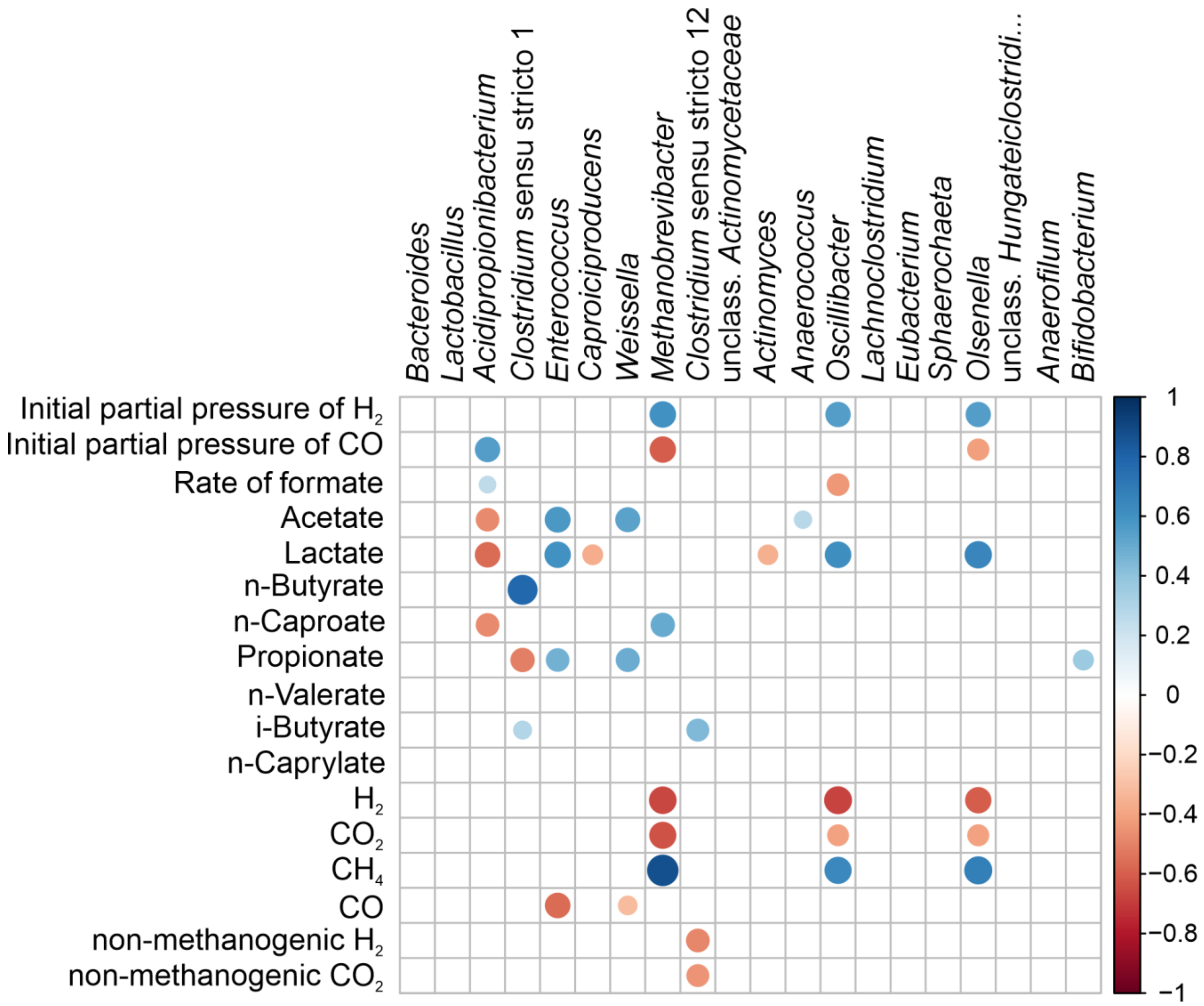

Figure S7. Spearman correlations between selected abiotic parameters and the relative abundances of the 20 most abundant genera in the dataset (n=38, p<0.01). Initial partial pressures of H_2 and CO and production (+) or consumption (−) rates of chemicals are shown.

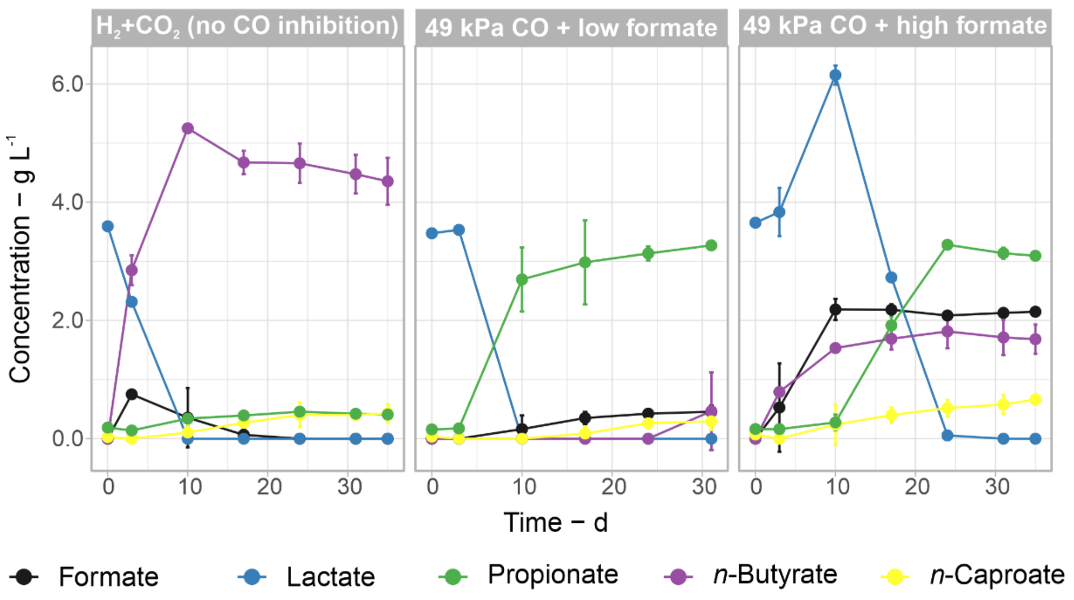

Figure S8. Fermentation profiles of the autochthonous community under a H_2+CO_2 headspace (uninhibited reference) and under a syngas headspace (49 kPa CO) with low and high formate accumulation. Mean values of duplicate bottles are shown. Error bars indicate standard errors.

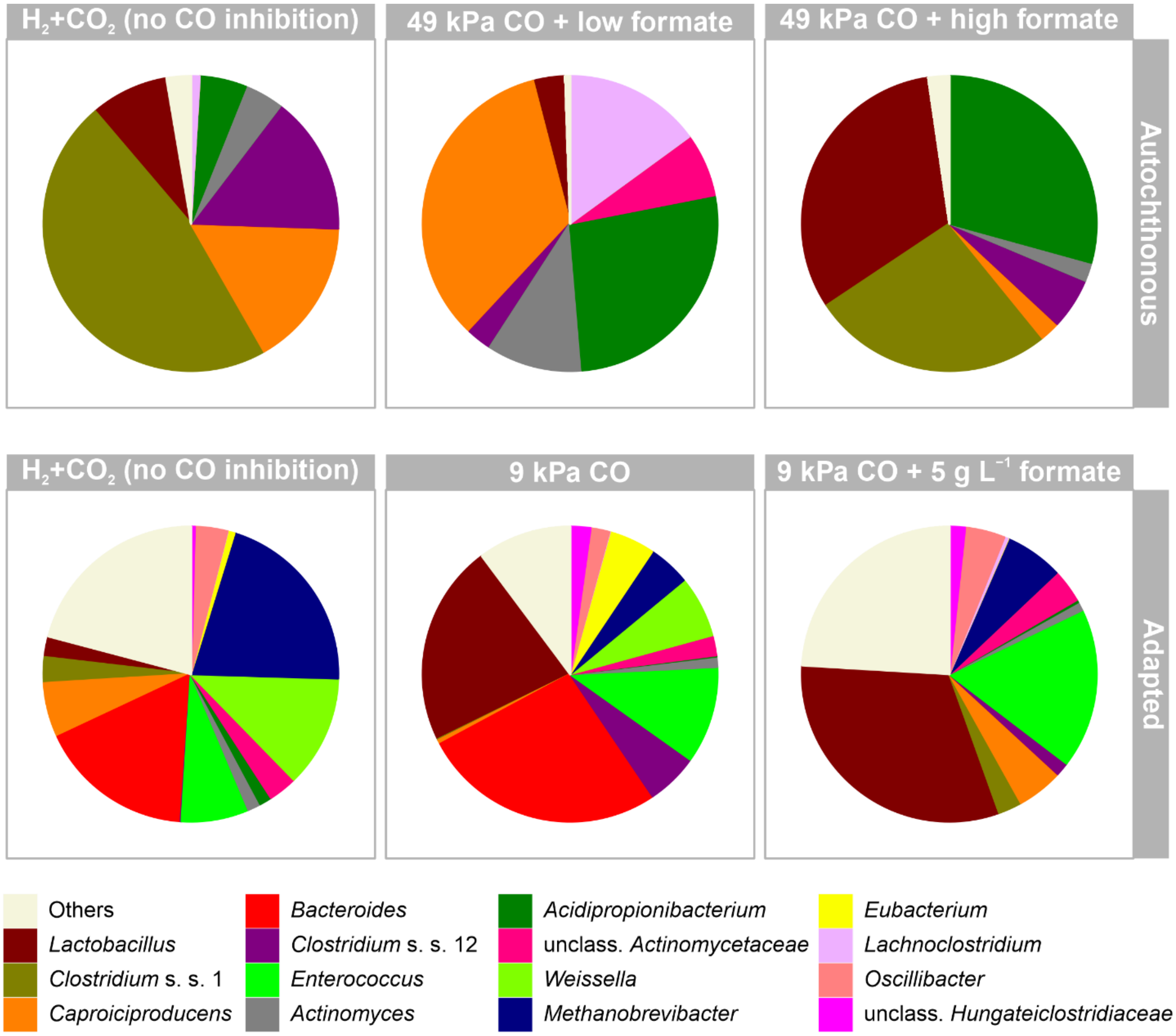

Figure S9. Composition of the autochthonous community and the syngas-adapted community depending on CO inhibition and formate availability. The 15 most abundant genera in the set are shown. S.s.: sensu stricto.
